# Death and the Oldest Old: Attitudes and Preferences for End-of-Life Care - Qualitative Research within a Population-Based Cohort Study

**DOI:** 10.1371/journal.pone.0150686

**Published:** 2016-04-05

**Authors:** Jane Fleming, Morag Farquhar, Cambridge City over-75s Cohort (CC75C) study collaboration, Carol Brayne, Stephen Barclay

**Affiliations:** 1 Cambridge Institute of Public Health, University of Cambridge, Cambridge, United Kingdom; 2 Department of Public Health and Primary Care, University of Cambridge, Cambridge, United Kingdom; 3 Primary Care Unit, Department of Public Health and Primary Care, University of Cambridge, Cambridge, United Kingdom; ISPO, ITALY

## Abstract

**Introduction:**

Increasing longevity means more people will be dying in very old age, but little is known about the preferences of the ‘oldest old’ regarding their care at the end of life.

**Aims:**

To understand very old people’s preferences regarding care towards the end of life and attitudes towards dying, to inform policy and practice.

**Methods:**

Qualitative data collection for n = 42 population-based cohort study participants aged 95–101 (88% women, 42% in long-term-care): topic-guided interviews with n = 33 participants and n = 39 proxy informants, most with both (n = 30: 4 jointly + separate interviews for 26 dyads).

**Results:**

Death was a part of life: these very old people mainly live day-to-day. Most were ready to die, reflecting their concerns regarding quality of life, being a nuisance, having nothing to live for and having lived long enough. Contrasting views were rare exceptions but voiced firmly. Most were not worried about death itself, but concerned more about the dying process and impacts on those left behind; a peaceful and pain-free death was a common ideal. Attitudes ranged from not wanting to think about death, through accepting its inevitable approach to longing for its release. Preferring to be made comfortable rather than have life-saving treatment if seriously ill, and wishing to avoid hospital, were commonly expressed views. There was little or no future planning, some consciously choosing not to. Uncertainty hampered end-of-life planning even when death was expected soon. Some stressed circumstances, such as severe dependency and others’ likely decision-making roles, would influence choices. Carers found these issues harder to raise but felt they would know their older relatives’ preferences, usually palliative care, although we found two discrepant views.

**Conclusions:**

This study’s rare data show ≥95-year-olds are willing to discuss dying and end-of-life care but seldom do. Formal documentation of wishes is extremely rare and may not be welcome. Although being “ready to die” and preferring a palliative approach predominated, these preferences cannot be assumed.

## Introduction

Living longer means dying older. Demographic changes mean more people are living their last years in very old age.[1] How does this affect their attitudes towards death, dying and care towards the end of life? The experiences and preferences of ‘older old’ people, or their carers, are rarely heard, but their voices are crucial to shaping end-of-life care services to reflect the priorities of increasingly older people approaching death.

The existing literature on end of life preferences and experiences of older people is limited, and particularly so in relation to very old people.[2;3] Research to date has typically sought bereaved relatives’ views,[4–8] though some pioneering studies with older people have been largely in long-term care settings,[9–13] occasionally with just community-dwelling older people,[14–18] but to our knowledge none so very old and none except our cohort[19] taking a population-based approach.

The present study informs policy, planning and practice by exploring thoughts on death and dying and preferences for end-of-life care with a representative sample of very old people—over-95-year-olds, their relatives and formal carers.

## Methods

A qualitative interview was added to the regular quantitative survey data collection at the 7^th^ wave follow-up (Year 21) of a longitudinal study of ageing, the Cambridge City over-75s Cohort (CC75C) study. Full study methods have are described elsewhere[20] (http://www.cc75c.group.cam.ac.uk/). The original population-based sample (n = 2166, 95% response rate) enrolled in 1985/87 using general practice lists have been re-interviewed every few years following Cambridge Research Ethics Committee approval and renewed consent. To avoid under-representation of the most frail, proxy informants were also interviewed. Interviews were conducted face-to-face in the interviewee’s usual home or occasionally, with proxies, by phone. Mortality was the main source of attrition.

In this Survey 7, following completion of the CC75C interviewer-administered structured questionnaire (http://www.cc75c.group.cam.ac.uk/pages/questionnaires), participants were invited to a further interview “more like a conversation than the usual survey” with the aim of understanding “what it is like to be so old, your experiences of care and your views on some of these issues that are more complex than a survey can explore.” A topic guide of open-ended questions (http://www.cc75c.group.cam.ac.uk/pages/additionaldata) explored issues participants had raised in past surveys, where quantitative methods had been inadequate to fully explore the depth of responses. When proxy informants were interviewed, they were asked what they believed would be the response from the older person as well as their own views and experiences. Multiple strategies maximised inclusion of these very old and often frail participants in qualitative data collection [see Box 1].

**Box 1. Multiple strategies used to source qualitative data.**

**Data sources:**

1. Transcribed audio-recordings of topic-guided interviews with study participants and proxies:
  - data usually collected at a second visit following the standard CC75C survey interview
  - data collected over several short visits for those with sensory or cognitive impairment
  - mixed method interview for those too frail to manage more than a single visit.
2. Transcribed extracts from audio-recordings of survey interviews with study participants and proxies where divergence from the structured CC75C questionnaire’s set response options provided relevant additional material.
3. Field notes for vignettes written up immediately after interviews
4. Additional information e.g. phone calls and correspondence from proxies.

To maximise the value of these data sources, the entirety of this relatively large qualitative archive was included in the analysis. A descriptive thematic analysis was conducted using a framework approach,[21] which facilitates working with large data sets, particularly where data collection is more structured, and where co-researchers share analysis. NVivo software (http://www.qsrinternational.com/products_nvivo.aspx) was used to manage and index the data prior to charting, mapping and interpretation. The analysis team, trained in framework analysis, consisted of two to three analysts at any one time (four individuals trained in qualitative methods) working independently, in parallel and together at various steps of the analytic process.

## Results

**Fig 1:**
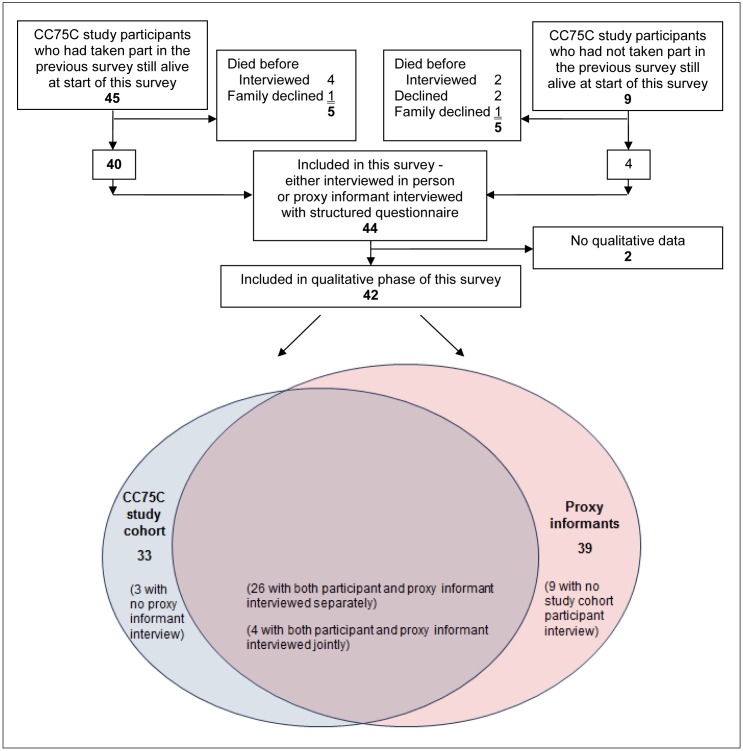
Attrition and participation in the CC75C study’s qualitative interview wave. The CC75C study’s population-based sample continued to be representative even in its Year 21 follow-up. Of n = 48 still alive 92% (n = 44) were included in this survey wave. Qualitative data collection for 95% (42/44) of these included recorded interviews with 79% (33/42) of these 95- to 101-year-old participants in person.

Of 54 study participants alive when Survey 7 started, 6 died before they could be interviewed. Of the 48 remaining, 44 (92%) took part in the usual survey. Qualitative data were collected for all but two people. For the majority (n = 30, 71%) both the participant and a proxy informant were interviewed, usually separately (n = 26) or for a few jointly (n = 4). For a small minority (n = 3, 7%) only the participant was interviewed and for one in five (n = 9, 21%) only proxy interviews were possible [see Fig 1]. All but six proxy informants were relatives, most commonly daughters (n = 20) or sons (n = 7); non-family included care-home staff, a live-in care-assistant and a friend. Participants were aged 95–101, mainly women (n = 37, 88%); 57% were community-dwelling (n = 20 in private dwellings, half of these living alone, n = 4 alone in sheltered accommodation) [see Table 1 for further demographics]. The full archive for qualitative analysis comprised 112 source documents (1269 pages) including anonymised transcripts from 95.5 hours of audio-recordings from 91 interviews relating to 42 older people.

**Table 1:** Demographic characteristics, cognition, disability and self-reported health of the study sample.

	*Total n = 42*	
**Age**	**Years**	(SD*)
*Mean Age*	97.5	(1.5)
*Median Age*	97.1	
*Interquartile Range*	96.2–98.4	
*Age Range*	95.4–101.4	
	**n**	**(%)**
**Sex**		
*Male*	5	(12)
*Female*	37	(88)
**Marital Status**		
*Married*	3	(7)
*Widowed*	36	(86)
*Separated/Divorced*	1	(2)
*Single*	2	(5)
**Education (by school leaving age)**		
*<15 years of age*	25	(60)
*≥15 years of age*	17	(40)
**Social Class By Occupation**^†^		
*Non-Manual*	22	(55)
*Manual*	19	(43)
**Accommodation**		
*Private house/flat*	20	(48)
*Sheltered housing*	4	(10)
*Residential home*	9	(21)
*Long stay hospital*	1	(2)
*Nursing home*	8	(19)
**Cognitive Function by MMSE** ^‡^		
*Normal cognition*	12	(29)
*Mildly Impaired*	6	(14)
*Moderately Impaired*	10	(24)
*Severely Impaired*	14	(33)
**Levels of ADL Disability** ^§^		
*No Disability*	6	(14)
*IADL Disability Only*	4	(10)
*IADL + PADL Disability*	30	(71)
**Self-reported Health** **		
*Very good/Good*	19	(66)
*Fair*	5	(17)
*Poor/Very poor*		
*Don’t Know/No Answer*	5	(17)
*Missing*	13	(31)

* ***SD*** = *standard deviation*

^**†**^ *Missing social class data for 1 respondent (2%) reflect missing data from baseline interview*

^**‡**^ **MMSE** = Mini Mental State Examination (score range 0–30), from which cognitive impairment was categorised as severe (0–17), moderate (18–21) and mild (22–25) or cognition was rated intact (26–30).

^**§**^ **ADL** = Activities of Daily Living, IADL = Instrumental Activities of Daily Living, PADL = Personal Activities of Daily Living.

Disability was defined as needing assistance in any 1 task.

**Self-reported Health is in comparison to others of same age.

Analyses yielded three main topics: 1) the context, beliefs and outlooks framing the lives of our very old study participants; 2) their attitudes towards dying and death; 3) their preferences concerning end-of-life care. Findings are presented below in sub-themes of each topic with sample comments (all names and identifying details have been changed) [see Box 2 for thematic overview].

**Box 2. Thematic overview.**

1. ***Context*, *beliefs & outlooks***
  1a) Everyone dying
  1b) Ready to die
  1c) Medicalisation postponing death
  1d) Euthanasia
  1e) Not ready to die
  1f) Outlook on life
2. ***Attitudes towards dying and death***
  2a) Thoughts about dying
  2b) Talking about death
  2c) Talking about funerals
  2d) The manner of dying
3. ***Preferences concerning end-of-life care***
  3a) Preferences regarding life-saving treatment
  3b) Admission to hospital at the end of life
  3c) Family members’ wishes for their relatives
  3d) Family members’ understanding of their older relatives’ preferences
  3e) Discussing end-of-life care preferences
  3f) Planning and documenting

### 1) Context, beliefs & outlooks

Participants vividly described the context in which they were living their later years, a context where contemporaries’ deaths heightened awareness of dying, though interviews revealed considerable variation in views on readiness to die or not, medicalisation of dying, euthanasia and life outlook.

#### 1a) Everyone dying

The age of these older people was so great that most of their contemporaries had died. Their circles of friends and family had diminished and death was a regular feature of life, leading some to question why they were still alive.

*“As people get older*, *as their friends die*, *there’s an element of ticking them off [laughing]”*

(2930-proxy)

One proxy measured this by the size of her mother’s Christmas card list–“*down to five now*”–and quoted her frequently asking “*for at least ten years ‘Why am I still here*? *All my friends have gone*.*’*”(1077-proxy)

However, there were positive outlooks. One proxy described her mother as a survivor, acknowledging deaths of others but able to carry on; others celebrated their survival:

*“…they became great buddies*. *And I do think she was sad when she died*, *but […] she didn't miss a beat […]*, *she carried on with her life*. *So I think there's obviously the survival thing there*.*”*

(3103-proxy)

Thus, for many, death and dying framed their outlook on their remaining lives. Some spoke, more negatively, of being on borrowed time:

*“Maybe she feels she’s on borrowed time*.*[…] She’s had underlying heart problems over time*, *so she must have had a sort of taste of her own mortality*.*”*

(142-proxy)

Most knew, and accepted, that they were going to die soon. Their lives had been long and were coming to an end.

*Daughter*: *We haven’t bought her new clothes for ten years*.*[…] ‘It’s not worth it*, *dear’ […] Same for the teeth*,*[…] she won’t do anything about her teeth […]*

*Son-in-law*: *I think the best one was the long life bulb that she gave our daughter ‘Something for you*, *it’s not worth me having a/*

*Daughter*: */a long life bulb*!*’*

(1077-proxy)

For some this was only a recent realisation, sometimes triggered by illness. A few indicated that, although towards the end of their life, they did not see the end as imminent:

*Interviewer*: *But if you think of your life as a journey*, *how do you feel about being at the particular spot where you are now in the journey*?

*Participant*: *Oh dear*, *that’s difficult*. *Well*, *I would say just over three-quarters of the way through*.

(3124)

#### 1b) Ready to die

Most felt ready to die, describing “waiting for it to happen”:

*“I’m ready to go*. *I just say I’m the lady in waiting […] waiting to go*.*”*

(645)

*“She doesn’t read anymore since her glasses were lost and she doesn’t want new ones*. *She’s waiting to die*.*”*

(3547-proxy)

Some linked this to quality of life:

*Interviewer*: *Would you say that you enjoy your life*?

*Participant*: *I’m past it*.

(2882)

Some felt they were a nuisance to others:

*“I wish I could snuff it […] I’m only in the way*.*”*

(2804)

Some were more desperate in their desire to reach the end, sometimes linking this to their longevity, or suggesting they had simply lived too long:

*“‘Please don’t let me live ‘til I’m a hundred’ she said*.*”*

(148-proxy)

Others described having nothing to live for and having had enough:

*Interviewer*: *How are you today*?

*Participant*: *[laughs] Lousy as usual […] I wish I wasn’t here*.

*Interviewer*: *[…] Why is that then*?

*Participant*: *Well*, *nothing to… nothing to live for*.

(1077)

Some had cried about it:

*“I wake up in the morning and think what the hell am I here for and I’ll cry*.*”*

(3346)

For others these feelings varied—they wished for death on bad days but on better days the will to live remained.

*“Some days she gets a bit fed up or cross*. *She'll say*, *‘Oh I wish I wasn't here*!*’ Or something like that […] And another day she's completely the opposite*.*”*

(3504-proxy)

#### 1c) Medicalisation postponing death

Well-intended medical interventions were viewed negatively as prolonging life by a handful of interviewees, an issue usually, but not exclusively, raised by proxy informants. One care home manager offered the explanation that *“in hospitals they do feel […] death is not something they want*, *so they will over-medicalise because it’s better than doing nothing”(142-proxy)*. She exemplified this with the ethical issues posed by enteral tube feeding decisions: “*It’s harder to take the thing out than to put one in*.”*(142-proxy)* However, relatives questioned the logic of some medical practices:

“*I was annoyed […] the doctor gave her her pneumonia injection […]—she had no quality of life… was ready to go […] [but] there was no consultation with us at all about it*. *[…] I was very angry with him*.”

(3403-proxy)

One 98-year-old couldn’t *“see any point in keeping people alive*”*(2999)*, confirmed in a separate interview with her niece: “*She thinks it’s absolute nonsense being kept alive*. *And that everybody here [care home] is being kept alive*. *[…] she will come out every now and then with […] ‘you look after us too well’*” *(2999-proxy)*. This was reiterated more strongly by a daughter whose mother thought it *“bloody silly keeping people [alive]”(1077-proxy)*.

#### 1d) Euthanasia

A step on from this, another daughter struggled with her perception that “*it seems to be society’s mentality that they keep you alive anyhow […] regardless*”*(2961-proxy)*. A son with similar doubts described a vivid memory of accompanying his mother to visit one of her friends with dementia following several strokes:

*“When she had her marbles*, *she said ‘Gordon*, *[…] if I ever get like that*, *for goodness sake put a*…*’*, *it was her words*, *not mine*, *‘put a pillow over my head*, *will you*?*’… But of course you can’t*. *If she was* compos mentis *enough to realise […] the state she was in*, *she would be very upset*.”

*(2916-proxy)*.

Another daughter said her mother would ask *‘why don’t they put me out of my misery*? *It’s pointless me lying here”(3211-proxy)*. One relative took a stern line in response to such comments, voicing a key dilemma:

*“I’ve had to be quite firm with her and said ‘Well*, *this is your lot*, *you have to accept it*. *[…] If you don’t commit suicide […] why put the responsibility of dying onto anybody else*? *That’s not fair*.*’”*

(2999-proxy)

Two daughters whose mothers had repeatedly expressed similar wishes considered potential legal repercussions and expressed doubts:

*“Someone would be assisting*, *wouldn’t they*, *if they gave her something to end her life*?*”*

(1162-proxy)

*“We’ve sort of been in sympathy with euthanasia […] [but] when it actually comes to that moment*, *to one’s nearest and dearest*, *it is actually another factor*. *I don’t know*.*”*

*(3124-proxy)*.

Another daughter raised assisted dying: *“If you offered her to go to Switzerland or Holland or wherever*, *she’d go*. *She’s been ready […] for a long time*.*”(1077-proxy)*, but recognised her mother’s ability to act on her wishes had diminished as her desire to die increased.

#### 1e) Not ready to die

However, for some the will to live remained strong:

*Interviewer*: *Do you sometimes think that life isn’t worth living*?

*Participant*: *No*. *I don’t think of things like that*.

(1110)

*“He’s not looking forward to not being here*. *He’s still got the life force very strongly*.*”*

(2930-proxy)

#### 1f) Outlook on remaining life

Many of the older people referred to “taking each day as it comes”, expressing thankfulness for where they were in life and content, at this stage, to take life one day at a time, not worrying too much about tomorrow. There was a sense of life ticking along until something drastic happened.

*“It is only day-from-day when you get to ninety-seven*.*”*

(3103)

Faith, or a lack of faith, provided a framework for end-of-life care preferences for some.

*Interviewer*: *How do you feel about the future*, *how do you think things will work out for you*?

*Participant*: *Well*, *being a Christian*, *I leave it to the Lord*, *yeah*, *what he wills*.

(1150)

*“I’m an atheist*, *or an agnostic*, *so I’ve no belief in a second*… *in a further life*. *I think it will be just as it was in the thousands of years before I was born*.*”*

(2930)

For some, however, their preferences for how they would like to die outweighed their beliefs:

*“She used to say ‘if I ever got cancer now*, *forget the chemotherapy and I wish there was something that I could be given to end my life peacefully’ […] And yet she was quite a religious woman*.*”*

(1162-proxy)

### 2) Attitudes towards dying and death

Thinking about death, talking about death and what might follow death emerged as separate themes in the interviews.

#### 2a) Thoughts about dying

As well as framing their thoughts on their remaining life, the deaths of others and faith perspectives provided a context for thoughts about death and dying. Most were not afraid of dying, either reporting it did not worry them or proxies saying they had not expressed any worries or fears about it:

*“I’m not afraid of dying […] at my age anything can happen*.*”*

(645)

For some this absence of fear was rooted in positive experiences of others’ dying:

*“When I look back in my family*, *my parents*. *They were alive*, *then they were dead*, *but it all went off as usual*. *Nothing really dramatic or anything […] Why should it be any different for me*?*”*

(1502)

But where the experience of others’ deaths had been negative, there was worry—not about death itself, but the process of dying:

*“[My aunt] found my mother’s end quite distressing*, *because she [pause] was not herself for quite a long time before she died*. *My mum was in hospital for a long time*, *and then in the home*. *[…] I think she worries that she’s going to be like that when the end comes*.*”*

(2882-proxy)

Worries about death also related to the impact death might have on others. This usually related to the emotional impact on the family or resulting loneliness for a remaining partner, but also to financial concerns.

*“The only thing I’m worried about is my sister*. *I hope that she’ll be not sad and be able to come to terms with it*.*”*

(1502)

“[…] he got awfully worried about money”

(3185-proxy)

A few reported thinking about death and dying, suggesting it was a worry, but for most it was something occasionally crossing their minds and not a great concern.

*“I suppose it comes across everybody… occasionally*, *but I wouldn’t make a mountain out of it”*

(1502)

*“Never given it much thought*.*”*

(2888)

There were some who stated explicitly that they didn’t think about, or preferred not to think about, or discuss, death. A care manager proxy suggested this was born out of fear.

“Some […] wouldn’t even broach the subject […] it was like tempting fate”

(142-proxy)

Death was welcomed where it was seen as a release. One older person said he was not afraid of death as it meant there was probably something wrong. Others felt death was both inevitable and approaching.

*“I’m not frightened to die*, *if that’s what you mean*. *No*, *not at all*. *I mean there isn’t any other future*.*”*

(3103)

The older people and their proxies seemed to accept this inevitability quite philosophically as beyond their control: *“Well*, *it’s no good making a fuss*. *If you can’t alter it*, *abide by it*.*”(1079)*

Despite the inevitability of death, its trigger, process and timing remained uncertain.

*Participant*: *[…] that’s if I go first*. *She may go first*.

*Sister*: *Well*, *it’s something we don’t know*, *isn’t it*? *[…] Just can’t say*.

*Participant*: *That is the reason*, *my love*, *for just letting it drift with the tide*.

(1502)

Death could be unpredictably sudden: *“[…] supposing I collapsed and went suddenly*? *I sort of always have a little bit of doubt at the back of my mind*.*”(1433)*

Or it could be a prolonged process involving a period of greater care need which might require planning, recognised as difficult without foresight:

*“But you can’t really plan the end of your life can you*? *How do I know if there’s gonna be someone here to look after me*.*”*

(1502)

For some their survival this long had itself been unpredictable:

*“[He] had said at [his] 80th ‘I shan't be here much longer‴(1516)* [now aged 99]

#### 2b) Talking about death

Proxies reported that death was rarely talked about:

*“That generation*, *they didn’t actually discuss death much*, *I don’t think*, *my parents*, *at all*.*”*

(1162-proxy)

Some described conversations that alluded to death but didn’t go as far as explicitly discussing it:

*“She doesn’t talk about dying*. *She just talks about it being time she wasn’t here [*…*] Which means the same*.*”*

(2882-proxy)

But a few described openly talking about death and the future.

*“[…] the other day […] she said*, *‘I should think I’ll snuff it soon*, *don’t you*?*’ I said*, *‘I don’t know*, *you tell me’ and she just laughs*. *I mean*, *she… You can laugh with her about it*, *you know*. *[…] She’s never morbid”*

(2804-proxy)

#### 2c) Talking about funerals

Discussion of funeral preferences was more common than talking about death, although the extent of discussions varied. Some had made their preferences clear:

*“Father […] said*, *‘Don’t forget I want to be buried up at the city cemetery’*. *And it came out in conversation that my mother […] wants to be cremated […] Father reminds us occasionally [*…*] ‘Don’t forget*!*’[laughs]”*

(1516+1523-proxy)

*“She’s told me where everything is and what I’ve got to do*. *She’s sorted all that out a long time ago*.*”*

(1540-proxy)

Some had made the plans themselves and paid for their funerals in advanced:

*“She’s written her little sort of obituary of herself and her life*. *[…] She did this a few years back*, *which is just as well she did before*…*”*

(1162-proxy)

*“I’ve paid for my funeral*.*[*…*]*, *did that several years go”*

(645)

Others had no preferences or were content to leave arrangements to their families:

*“I can quite safely leave it with them to make arrangements*.*”*

(1150)

Some had simply not discussed it:

*Interviewer*: *Has she talked to you about the funeral*?

*Participant*: *No*. *No*. *No*. *No*, *it’s not something I’d bother her with*, *because […] Mum knows that I’d do what she would want anyway*.

(2804-proxy)

Others noted the difficulties of discussing funerals:

*“I did ask her when she wasn’t well*. *I said*, *“Do you think you would want to be buried or cremated*?*” And she said*, *‘Well*, *cremated*, *I think*.*’[…] […] I find it very difficult talking to her*. *I don’t feel that I can*. *You see*, *I say to my children ‘Oh*, *that’s a nice song on the radio*. *Oh*, *perhaps I’ll have that at my funeral’ [laughter]*. *And you couldn’t to my mum”*

(1079-proxy)

#### 2d) The manner of dying

The manner of death was of more concern than its imminence. Although some said they had not really thought about dying, many explicitly expressed the wish to die peacefully, pain free and preferably while asleep—to *“just slip away quietly*.*”(3194)* Indeed death coming suddenly was seen as positive:

*“I’d be quite happy if I went [snaps fingers] suddenly like that*.*”*

(3403)

Relatives echoed the same wishes for their loved ones:

*“I just hope that she doesn’t get a […] catastrophic illness that she’s going to suffer*. *I would just like her to sleep one day*, *you know*, *because I don’t want her to have to go through anything else*.*”*

(2888-proxy)

*“I think I would love […] her to live as long as she feels she wants to*. *And then I would love her to keel over*. *I mean… Wouldn’t that be wonderful for all of us*?*”*

(3124-proxy)

### 3) Preferences concerning end-of-life care

By and large, interviewees talked readily about end-of-life care preferences.

#### 3a) Preferences regarding life-saving treatment

When asked whether, if they had a life-threatening illness, they would “want to receive treatment that would save [your] life or prefer treatment that would just make [you] comfortable”, preferences for life-saving treatment were unusual and even tentative:

*Interviewer*: *[…] would you want the doctors to go all out with treatment and so on*?

*Participant*: *Well yes*, *I should think so*. *Yes*.

(1110)

“Make me comfortable”(2952) was a far more typical response and proxy informants tended to echo the older people’s dominant preference for comfort rather than life-saving treatment:

*“I would say she would just like to be made comfortable*.*”*

(1540-proxy)

The term “life-threatening” in our questioning signified to some that quality of life would be threatened, prompting qualifying responses: *“I wouldn’t want a thing like these terrible things where people go on living and deteriorating”(2930)*. Some felt their preference would very much depend on specific circumstances.

“…whether it could be treatment that I could get about or I had to be bedridden”

(1079)

*“I haven’t thought about it*. *It’s a decision you can’t make unless you’re in that position*.*”*

(3103)

Relatives were similarly aware of the complexity of the uncertainties.

*“Well*, *that’s a difficult question*, *because it’s all according to how she would be*. *[…] when you get to that stage*, *to resuscitate somebody at 96 I think is a bit odd*. *But*, *on the other hand*, *my mother has quite a strong spirit*, *[…] she would probably want that*.*” […] [addressing mother] “If you had a bad stroke*, *mother*, *and I knew you were gonna be paralysed*, *and you couldn’t think for yourself*, *and they said to me ‘Shall we resuscitate your mother*?*’*, *I would say ‘No*.*’”*

(645-proxy)

Older people were fatalistic about decisions being made by others.

*“Well*, *whatever’s thought necessary*.*”*

(1433)

Some stated that they would leave such decisions to others—relatives, health professionals or even God.

*“Her son’s been taking the decisions for the last ten*, *fifteen years perhaps*.*[…] That’s just […] yet one more decision that he will take*, *because she’s just handed decisions over to him*.*”*

(3403-proxy)

*“I never thought about that*. *I think it should be left to the doctor*.*[…] Well*, *what do we have doctors for if when anything happens we drop them*?*”*

(2888)

*Interviewer*: *And you’ve talked a bit about […] religion and feeling that it’s in God’s hands*, *what happens from now on*.

*Participant*: *Yes*, *yes*.

*Interviewer*: *Is that something that you think about quite a bit*?

*Participant*: *No*, *it’s just part of my faith*. *I can just leave it with confidence*.

(1150)

Trust in those to whom decision-making is passed was considered crucial.

*“He trusts his doctor enough to know that his doctor wouldn’t advise it unless it was essential […] so if his doctor said he had to*, *he would go [to hospital] because he trusts […] him*, *and we would too*. *[…] That’s very important*.*”*

(2930-proxy)

It tended to be older people themselves who said they had discussed their preferences:

*“My family know my feelings about Alzheimer’s*. *They do know that*. *The doctor knows it*.*”*

(1433)

*“I think they would know my views on that*.*[…] which would not be to continue life*, *[…] I once mentioned to my doctor […] and he said ‘Well*, *I’ve made a note on your file saying* no heroics.*’”*

(2930)

It was relatives who reported never having had any such discussion:

*“She hasn’t said […] I don’t really know*.*”*

(1110-proxy)

*“We haven’t discussed that at all*. *[…] And I couldn’t put words into her mouth I don’t think*.*[…] And of course when it does happen it’s possibly too late to think about it*.*”*

(1111-proxy)

#### 3b) Admission to hospital at the end of life

Asking about life-saving treatment often elicited views, mostly negative, regarding hospital admission. Only one woman said she had “*not given it any thought*”(148) and one daughter thought her mother would positively welcome it: *“That’s where she thinks you get proper care*.*”(1077-proxy)*. The minority happy to go to hospital qualified this with particular circumstances: *“If I had an accident or something like that*.*”(1110)*. Most were quite adamant about not wanting this–*“I should hate it […] I just wouldn’t like it*.*”(2916)–but* rarely gave reasons, though one care home manager explained:

*“If she went to [hospital] quite poorly*, *I think she would be full of anxiety and I think it would exacerbate any illness she had*. *I think she would find it alarming actually*.*”*

(3103-proxy)

Many older people recognised hospital admission might arise despite preferences against this: *“Well*, *I’d have to go I suppose”(1516)*. This was sometimes voiced in tones of acceptance, albeit reluctant:

*“It would be selfish not to go somewhere where I can be looked after*. *It’s not fair to them […] I would have to accept it […] shouldn’t really like it*. *[I’d rather] stay put*.*”*

(1079)

Others were more pragmatic; whether someone was admitted or not was occasionally viewed as neither here nor there:

*“If they want me in the hospital*, *I suppose they’ll take me*. *[…] I think you’re beyond caring”*

(1077)

Family members were often aware of preferences, one niece linking this with her aunt not wanting to go into long-term-care either:

*“She's dead against going*. *[…] She doesn't like hospitals*. *She doesn't want to go*. *[…] I think she thinks […] to go to bed and go to sleep […] without going into hospital*, *I think that's her ideal*, *just dying in her own home*. *[*…*] I do know that she's so against going into a home or hospital*.*”*

(2952-proxy)

Another used the phrase *“I wouldn’t put her in hospital anymore”(148-proxy)*, often heard in relation to moving older relatives into care.

#### 3c) Family members’ wishes for their relatives

There were clear parallels between what the proxy informants tended to say they thought their older relatives wanted, as described above, and what they wished for on behalf of their older relatives. Very similar themes emerged as their most common hopes—avoiding any suffering, hospital admission or prolonging of life, and achieving a peaceful death at home or in a familiar care home.

*“Kept comfortable*. *That’s what I would want for her really*.*”*

(2804-proxy)

Relatives echoed the older participants’ awareness that, regardless of preferences, decision-making may be taken over by others:

*“I mean*, *in the end it may ultimately be our decision anyhow*, *rather than hers”*

(2961-proxy)

Only one proxy informant, not a relative but a care home manager, expressed more general views about the manner and place of death:

*“To die in the home she regards as home is definitely more dignified and gentler*, *because she is surrounded by people she knows*.*”*

(3103-proxy)

#### 3d) Family members’ understanding of their older relatives’ preferences

Occasionally a relative explicitly conveyed the difficulty of understanding how their elderly parent felt:

*“I wonder sometimes if she wants to live to 100*. *[…] to say that she’s done it*, *you know*.*”*

(1079-proxy)

A few mentioned views expressed in the past, for example before dementia had limited the ability to discuss preferences:

*“Knowing Mum’s opinion from the past*, *I said I thought it would be kinder just let her go without this extra effort of resuscitating the heart and so on*.*”*

(3149-proxy)

Far more commonly family and paid carers commented that the older person they cared for had never specifically discussed care preferences with them.

*“She knows she's going to die one day*. *I mean*, *we all do*. *But she never really mentions saying that she wants to go here or any of that*.*”*

(3504-proxy)

However, by far the majority felt they knew their relative well enough to predict their views:

“*I would expect […] she would do what [her sisters] have all done*.*[…] and that’s just take water*.*[…] When […] life had become too much of a burden […] they just put themselves onto water only until they faded away*. *And I would expect that she would probably*, *if she had the nouse*, *do that*.*”*

(2999-proxy)

Most tended to voice what they understood the older person’s preferences to be as definitive statements, rarely phrased with any uncertainty:

*“She doesn't believe in a lot of highfaluting interventions*.*[…] Nature will take its course*. *And if it is on the cards that any particular infection makes her shuffle off her mortal coil then that is the way it is supposed to be*.*”*

(3103-proxy)

Again these conveyed a clear preference for palliative care, rather than life-saving interventions, and a particular abhorrence of severe dependency:

*“She wouldn’t*… *wish the life support machine to be switched on[…] to be resuscitated*, *because she’s been saying now for so long that she wants to go*. *I mean what is there for her*? *She’s got nothing*, *has she*?*[…] Her quality of life is zilch really*.*”*

(1077-proxy)

In a couple of noteworthy instances there was marked contrast between what older people said and what their relatives thought would be their views. One daughter said *“I don’t think she’d really and truly want to be resuscitated”(1110-proxy)*, whereas her mother was one of the minority in favour of active treatment (see 3a) above). Another daughter thought both her parents would “*like treatment to make them comfortable rather than life-saving*”*(1516+1523-proxy)*, though they hadn’t discussed it, but her mother in fact wanted to “*have treatment as long as [she] could*.”*(1523)*.

#### 3e) Discussing end-of-life care preferences

A few examples were mentioned of how older people had discussed, or would like to discuss, their end of life preferences. One woman and her niece wanted the care agency to be “*aware of [her] policy decision”* that she *“would rather be nursed at home”(3154-proxy*), and a wife described how her husband raised his concerns with their GP:

*“When Leonard first came back and heard he had this other cancer Dr Warner came to see him and Leonard said to him ‘Two things I want*, *please*, *Andrew*, *is stay at home and if I’m in pain*, *would you try [*…*] if you could keep me out of pain*. *And I want you to promise me*.*’ And Andrew Warner was very sweet and he said ‘Leonard*, *I can’t promise*, *but I will do my very best*.*’”*

(3185-proxy)

These discussions were usually with care professionals, but a care home manager commented that it was not necessarily with older people themselves that professionals broached the topic:

*“Historically what happens in hospitals is they tend to*… *if it’s not asked beforehand they go straight to the relatives*. *They won’t broach it with the individual*, *which is actually […] against the Data Protection Act*, *and it’s nobody else’s business*. *But it is how the culture of hospitals works unfortunately*.*”*

(142-proxy)

However this care home manager pointed out not all her residents wished to have such discussions:

*“Each individual person deals with it differently*. *Some people do feel that they’ve lived a decent age and they’re not too worried about it*, *and other people have a great fear of death and even in their late nineties would be absolutely terrified*. *So I think that’s an individual thing*.*”*

(142-proxy)

She had found that a variety of approaches could be useful:

*“I’ve spoken to the GP and we were talking about how we open those conversations*, *and we were looking for perhaps some literature*, *rather than just to say 'Oh well*, *by the way…'[laughs]*, *because I’m worried because old people may feel that*… *is it a loaded question*? *Is there supposed to be a way they’re supposed to answer because are they a burden now and all this sort of stuff that sometimes people start feeling*. *[…]We are trying to get it as a group discussion so they don’t feel it’s something they have to decide on themselves*. *We could open it up […]as a general discussion*. *How do they feel about it*? *I think it’s the way forward*.*”*

(142-proxy)

Her long experience pointed to the practical difficulties of having conversations about end-of-life care preferences:

*“I’ve not had long discussions*, *because of [her] hearing problem […] but I would hope if we can get this hearing aid sorted that would give her an outlet to join those discussions*. *It’s very difficult to write them down on paper […] And she’s got to shout the answer back at you and you’ve got to re-clarify it*.*”*

(142-proxy)

This experience provided considerable insights into potential pitfalls. The timing of these conversations was felt to be particularly crucial and often problematic, especially given that views may change:

*“It’s nice to elicit a conversation*, *but it can be quite scary*. *I could imagine if I said ‘If I have a dense stroke tomorrow*, *leave me’*, *but then if I had the dense stroke tomorrow I may decide… you know*, *it is a worry*.*”*

(142-proxy)

Within families it appeared there was very rarely any specific discussion:

*“We haven’t discussed it in so many words*, *but by the way it’s come into conversation here and there… She has talked about upstairs (long-term-care)[…] Once I got across to her that […] it was if you weren’t very well[…] you were upstairs […] I think in a way that’s set her mind at rest that she won’t have to go into hospital*.*”*

*(2882-proxy)*.

One relative commented that the study interview was making her think it was something she needed to bring up. She felt unsure about how to raise it and who in the family might best do this: *(1150-proxy)*.

“*Maybe something happening to someone else will trigger that conversation*.*[…] His doctor son could introduce it in relation to his work*.*[…] It’s quite possible that something could happen*. *He could have a stroke[…] And it would be out of the blue and we would not be prepared necessarily*.*[…] I’ll mention this to Desmond actually*. *Does he know his dad’s feelings on this*? *[…]just opened a little door in my own mind*.*”*

(1150-proxy)

#### 3f) Planning and documenting

If plans for the future were mentioned, it was generally in the context of *not* making plans:

*“I don’t*. *I just let it come*.*”*

(2952)

*“No*, *I haven’t made any plans*, *because I’m not ill*.*”*

(3194)

Putting preferences in writing was rarely mentioned. One woman told her daughter that she should put what she wanted in writing so that if she were *“suddenly taken ill and I was very ill*, *and the doctor said to you ‘Well*, *what do you want me to do*?*’ you’d know what I wanted”*. Her daughter agreed, although she felt this was a “*weird […] decision*.*”(645 & 645-proxy)*

‘Living wills’ only came up with one family: the participant’s daughter explained that her father would never sign a living will:

*“He’s talked quite firmly that he would never do that*. *[…] I think it’s the logical*, *the rational in him that says*… *[…] that people change their mind and he wouldn’t wish to tie himself”*

(2930-proxy)

The only example of when specific wishes had been documented was in a care home where:

*“We now have it written in her notes that she’s not to go to the district hospital […] and we have a wonderful little cottage hospital in Easingstoke*, *with about fourteen*, *fifteen beds which everybody longs to go to*. *You know*, *it is just super […] lovely reputation*.*”*

(2999-proxy)

However, these recorded wishes had then been overlooked by agency nursing staff who called an ambulance when concerned about the resident’s condition one night.

## Discussion, Implications and Conclusions

We sought thoughts on death and dying and preferences for end-of-life care with a representative sample of very old people, their relatives and formal carers. This rare dataset of qualitative interviews described ≥95-year-olds’ lives as contextualised by death, with the majority wondering why they were still there and a minority celebrating their survival. Death was now a part of life for these very old people who were mainly living day-to-day. Most were ready to die, reflecting their concerns for quality of life, not being a nuisance, having nothing to live for and feelings of having lived long enough. Contrasting views were rare exceptions but voiced firmly. Unwanted medical prolongation of life was an issue for some and mention of euthanasia related to loss of quality of life, usually raised by proxy informants for whom legal implications were a concern. Most were not worried about death itself. Concerns, coloured by experience of others’ dying, were more about impact on those left behind and the dying process; a peaceful and pain-free death, preferably during sleep, was a common ideal. The wide variation in attitudes ranged from not wanting to think about death, through accepting it as inevitable and approaching, to welcoming or even longing for its release. Preferring to be made comfortable rather than have life-saving treatment if seriously ill, and wishing to avoid going into hospital, were both commonly expressed views, again with a few firm exceptions. Some stressed choices would be affected by circumstances, such as likelihood of severe dependency. They fatalistically accepted decisions might well be taken by others, family and healthcare professionals, and trust in decision-makers was important. Some felt their destiny lay with God or fate. Uncertainty hampered end-of-life planning even when death was expected soon. There was little or no planning for the future, some consciously choosing not to. Written documentation of wishes was virtually unheard of, except sometimes for funerals or wills; the only mention of living wills was in relation to a reluctance to prepare one, and the only example we came across of an advance care plan, in nursing home notes, had been ignored. Despite a notable willingness to discuss end-of-life preferences in most interviews, and death being so ubiquitous, previously having talked openly about death was uncommon, often only alluded to or couched in humour. Not all the older people wanted to have such discussions; for the minority who had, these explicit discussions had usually been with care professionals. Families and care professionals found these issues hard to raise, generally saying preferences had never been discussed. They felt they knew their older relatives well enough to predict their preferences, usually palliative care, mirroring what most older participants wanted, although we found two discrepant views.

Rarely are the views of the very old heard. Our sizeable qualitative dataset uniquely evidences the perspectives of a representative sample of over-95-year-olds. In contrast to much qualitative research that aims to capture diversity through purposive sampling, these were population-based cohort participants and moreover were well-characterised by the mixed methods CC75C study’s quantitative epidemiology. Although a single-centre study, sample characteristics are in line with national data available for this age-group and other developed countries. Non-response potentially limits research validity, but 79% of the surviving cohort were interviewed in person and proxy interviews maximised participation to 92%, ensuring people across the cognitive and health spectrum were included. Proxy informants frequently quoted participants themselves, but there are inevitable uncertainties regarding the validity of perspectives heard second-hand. Reliance on informants to represent older relatives’ preferences, as we found most could, is not only a research issue; the occasional discrepant views we found have implications for practice, highlighted in other studies too.[22–24] However, including these reported views, despite the limitations of proxy reporting, ensured we captured some of the voices of those least able to express themselves. A further limitation was the study’s inability to explore how very old peoples’ views might change over time, particularly as they neared death, a question of importance given their unpredictable circumstances that merits further research.

Our study adds to the few studies to date that have sought the views of older people themselves,[9–19;24] rather than just through relatives.[4–8] Although previous research generally interviewed younger old people, we found overlapping themes, also highlighted in the few relevant reviews.[2;3;10] Heterogeneity of views is unsurprising,[2;3;25;26] but some common themes are striking: even in their late 90s or beyond our participants generally conveyed a ‘living for today’ approach previously reported[2;12–14;27] and comfortable acceptance of death.[2;3;26] Others too have found a readiness to talk about dying and end-of-life care,[2;3;11;16] though this rarely happened,[2;12] and willingness to make plans was less clear-cut[3;16;28]: one UK study found people living alone in their 80s “wanting to prepare for and to have a choice with regard to where and when they might die”,[15] but a US study found housebound older people were reluctant to plan for serious illness or dying, especially resisting decision-making on hypothetical future dilemmas, preferring to wait till they “cross that bridge”.[14] Other studies of people with dementia and their family carers have also reported the perceived difficulty of making formal plans for unknown futures.[24;27] Other researchers found, as we did, a minority who preferred not to discuss dying at all[3;14] and an expectation by some that others might make decisions when necessary.[3;11;12] As in our study, generally people were more likely to plan for death—funerals or wills—than dying,[2;11;14] although only a minority worried about death itself, most were more likely worried about the manner of dying.[15;19]

Our study found that an over-whelming majority of our very old sample had strong preferences for palliative care–“being made comfortable”–versus “life-saving treatment”. Others have also reported a majority preferring not to have interventions[11] and found preferences for life-sustaining treatment declined with decreasing physical or psychological functioning.[29] Some questioned the value of intervention: “Why are we being kept alive?”[18] Although only a minority wanted life-saving treatment, these were strong preferences but had not been communicated. Although current policy encourages early discussion and documentation of end-of-life care preferences,[30–32] our study and others illustrate how multiple barriers to advance care planning[3;8;24;33;34] apply also to other relevant options rarely discussed, such as moving into long-term care,[35] not least how unpredictability and changing contexts may change choices.[9;11;14;23;29;36–40] Others have given thoughtful consideration to the need to recognise that more dying people are following longer frailty trajectories and the implications for care services.[6;16;26;41;42] Previous research has highlighted challenges of improving communication about preferences[7;25;26;34;40;41;43–46]; we noted with interest other researchers’ comments that participants welcomed interviews as an opportunity to express thoughts,[11] as did many of ours. We found questions on specific topics could open up dialogue with people hesitant to discuss dying in general. Interviewees themselves suggested that “being surrounded by death” provided opportunities to start conversations, and both family and professional carers admitted being hesitant to broach the matter, at times leaving an older person with concerns and information needs, for instance pain relief, that no-one addressed.

We and others have described the complex transitions often experienced by very old people approaching the end of life,[38;47–52] and there is widespread recognition that transfers between care settings may be detrimental.[42;53–55] Most of our participants viewed the prospect of hospital negatively, but supporting older people to achieve current preferences in changing circumstances is a major challenge for research and practice. Prognostication uncertainties in frailty trajectories make determining when hospital admission is ‘inappropriate’ challenging, and too much ‘admissions avoidance’ risks denying very old people diagnosis, treatment and care that may be entirely appropriate.[38;42;56] Half of UK deaths aged ≥85 are in hospitals and a third in care homes[53] so improving end-of-life care for very old people in all settings is a priority.[7;30;41;42;54;57–60] Prospective population-based research with older old people, and their informal and formal carers, examining determinants of staying-in-place or transitions is needed to understand what would enable older people to die in the place and with the support they would choose.
